# Integrative description of a new *Dactylobiotus* (Eutardigrada: Parachela) from Antarctica that reveals an intraspecific variation in tardigrade egg morphology

**DOI:** 10.1038/s41598-020-65573-1

**Published:** 2020-06-04

**Authors:** Ji-Hoon Kihm, Sanghee Kim, Sandra J. McInnes, Krzysztof Zawierucha, Hyun Soo Rho, Pilmo Kang, Tae-Yoon S. Park

**Affiliations:** 1 Division of Polar Earth-System Sciences, Korea Polar Research Institute, 26 Songdomirae-ro, Yeonsu-gu, 21990 Incheon, Korea; 2 Polar Science, University of Science & Technology, 217 Gajeong-ro, Yuseong-gu, 34113 Daejeon, Korea; 3 Division of Polar Life Sciences, Korea Polar Research Institute, 26 Songdomirae-ro, Yeonsu-gu, 21990 Incheon, Korea; 4 British Antarctic Survey, Natural Environment Research Council, High Cross, Madingley Road, Cambridge, CB3 0ET UK; 5 Department of Animal Taxonomy and Ecology, Faculty of Biology, Adam Mickiewicz University, Poznań, Uniwersytetu Poznańskiego 6, 61-614 Poznań, Poland; 6 East Sea Environment Research Center, East Sea Research Institute, Korea Institute of Ocean Science & Technology, 48 Haeyanggwahak-gil, Uljin, 36315 Gyeongsangbuk-do Korea

**Keywords:** Animal physiology, Biodiversity

## Abstract

Tardigrades constitute one of the most important group in the challenging Antarctic terrestrial ecosystem. Living in various habitats, tardigrades play major roles as consumers and decomposers in the trophic networks of Antarctic terrestrial and freshwater environments; yet we still know little about their biodiversity. The Eutardigrada is a species rich class, for which the eggshell morphology is one of the key morphological characters. Tardigrade egg morphology shows a diverse appearance, and it is known that, despite rare, intraspecific variation is caused by seasonality, epigenetics, and external environmental conditions. Here we report *Dactylobiotus ovimutans* sp. nov. from King George Island, Antarctica. Interestingly, we observed a range of eggshell morphologies from the new species, although the population was cultured under controlled laboratory condition. Thus, seasonality, environmental conditions, and food source are eliminated, leaving an epigenetic factor as a main cause for variability in this case.

## Introduction

Phylum Tardigrada is a microscopic metazoan group, characterized by having four pairs of legs usually terminated with claws, and is considered to be related to the arthropods and onychophorans^1^. They have attracted attention due to their cryptobiotic ability^2–7^, which helps them to occupy a variety of habitats throughout the world, including the harsh environments of Antarctica. The challenging environments of Antarctica are represented by a depauperate biodiversity, in which tardigrades have become one of the dominant invertebrate groups^8–13^. Around 60 tardigrade species are recorded from Antarctic, with the Antarctic Peninsula having the most rich diversity^14–16^. In comparison to the much smaller north polar areas, such as Alaska, Svalbard, or Faroe Island^17–20^, the biodiversity of Antarctic tardigrades is significantly underestimated.

King George Island (KGI), the largest in the South Shetland Archipelago, covers about 1300 square kilometres comprising a mountainous landscape that is largely ice-covered with pockets of ice-free coastal regions^21,22^. Eight countries operate permanent research bases, with most established at the western end, between Fildes Peninsula and Admiralty Bay. This makes KGI one of the most well studied regions of Antarctica (e.g. refs. ^23–26^). Compared to the recent studies devoted to marine meiofauna around KGI (see: refs. ^27–29^), studies on KGI terrestrial invertebrates have received little attention^30–32^, with rather outdated species lists (e.g. ref. ^33^), and even more so for tardigrades^34–36^.

Since tardigrades have a limited suite of taxonomic characters, eggshell morphology is considered an important trait for those groups that lay ornamented eggs^37^. For example, *Dactylobiotus dispar* (Murray, 1907)^38^ and *Dac. octavi* Guidetti, Altiero and Hansen, 2006^39^ have extremely similar adult morphology, but their distinct egg morphologies aid differentiation of the two species^39^. Interestingly, intraspecific variation in egg morphology is reported in some species. Differences in lineages, life modes, or differential gene expressions have been suggested for the possible cause of the variation (e.g. refs. ^40–43^), but no clear answer has been provided.

*Dactylobiotus* Schuster, 1980^44^ (hereafter referred as *Dac*.^45^) is a freshwater genus occurring worldwide (see: ref. ^46^) including both polar regions^33,39^. Only one species, *Dac. ambiguus* (Murray, 1907)^38^ has been documented from islands near the Antarctic Peninsula, including KGI^33^, Signy Island^47^, and Alexander Island^48^. The eggs from these studies were described to be significantly different to the eggs of nominal *Dac. ambiguus* from the type locale in Scotland^38^, or records from Canada^49^. A new species *Dac. caldarellai* Pilato and Binda, 1994^50^ was established based on two adult specimens from Tierra del Fuego, Chile, and their claw morphology was comparable with the description of *Dac. ambiguus* reported from KGI by Dastych^34^. Accordingly, it was suggested that the eggs from KGI, as recorded by Dastych^34^, would also belong to *Dac. caldarella*^*i*50^. However, this suggestion needs verification, since no eggs of *Dac. caldarellai* have been reported from the type location (Tierra del Fuego)^50^.

Here we report the morphology, morphometry, and partial molecular sequences of three genes (a small ribosome subunit (18S rRNA), a large ribosome subunit (28S rRNA), and cytochrome oxidase *c* subunit I (COI)) of a new species *Dac. ovimutans* sp. nov. from KGI, Antarctica, which shows an intraspecific variation in the egg morphology. Since the specimens of the new species were cultured under laboratory condition, the cause of the morphological variation in the eggs can be also explored.

## Results

-Taxonomic account

**Phylum:** Tardigrada Doyère, 1840^51^

**Class:** Eutardigrada Richters, 1926^52^

**Order:** Parachela Schuster, Nelson, Grigarick and Christenberry, 1980^44^

**Superfamily:** Macrobiotoidea Thulin, 1928 in Marley, McInnes and Sands, 2011^53^

**Family:** Murrayidae Guidetti, Rebecchi and Bertolani, 2000^54^

**Genus:** *Dactylobiotus* Schuster, 1980^44^

**Type species:** *Dactylobiotus grandipes* (Schuster, Toftner and Grigarick, 1977)^55^

*Dactylobiotus ovimutans* sp. nov.

Material examined: Holotype (slide label: *Dactylobiotus* Sejong 044), reproduced from a population collected from the Lake Critical Zone Observatory (CZO), Barton Peninsula, King George Island, South Shetland Islands (62°14′24.140"S, 58°44′36.571"W, see: Supplementary Fig. S1), coll. Sanghee Kim. Details for locality of 60 paratypes (slide labels *Dactylobiotus* Sejong 001–043 and 045–061) and 59 eggs (slide labels *Dactylobiotus* Sejong egg 001–059) as mentioned above. Additional paratypes used for SEM (Scanning Electron Microscope) analysis: 9 paratypes (*Dactylobiotus* stub 01) and 14 eggs (*Dactylobiotus* egg stub 01).

### Description

**Figure 1:**
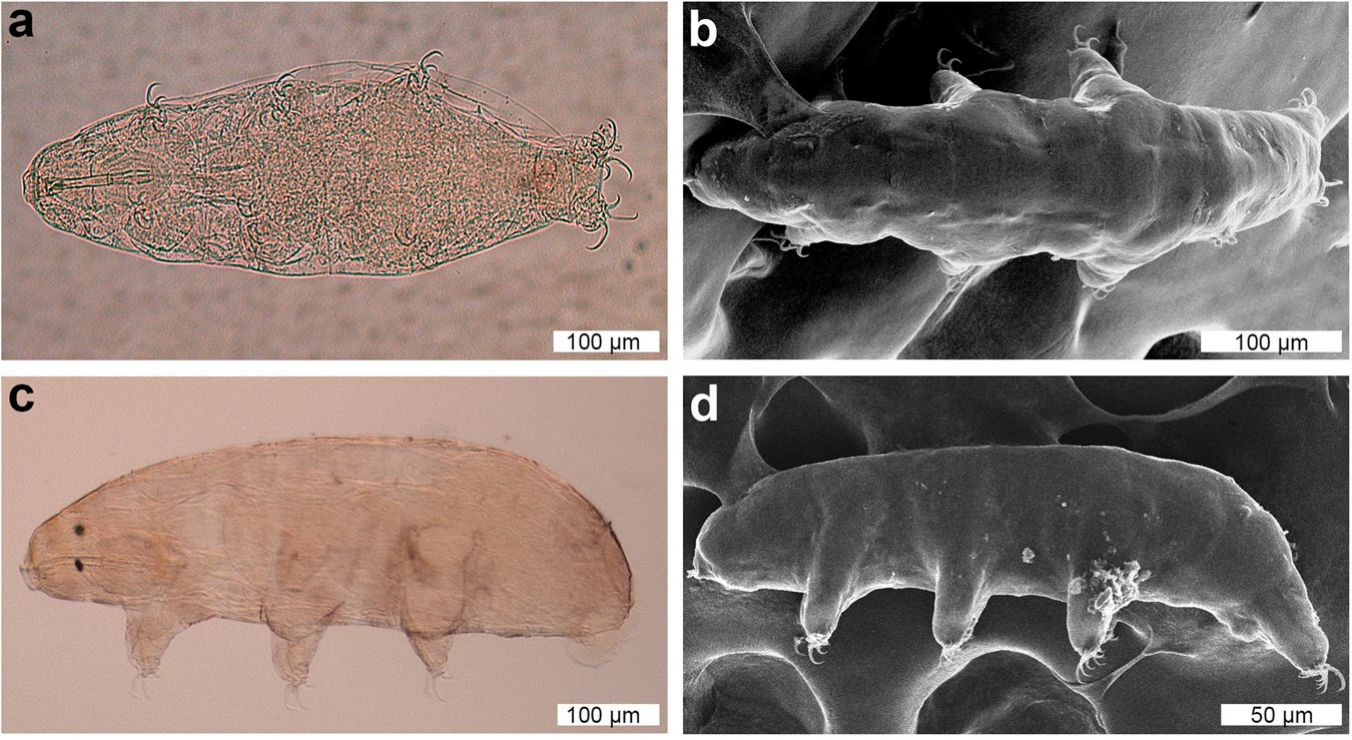
DIC and SEM images of *Dactylobiotus ovimutans* sp. nov. (**a**) The entire body of the holotype (DIC). (**b–d**) The paratypes. (**b**) The dorsal view (SEM). (**c,d**) The lateral view (DIC and SEM respectively).

**Figure 2:**
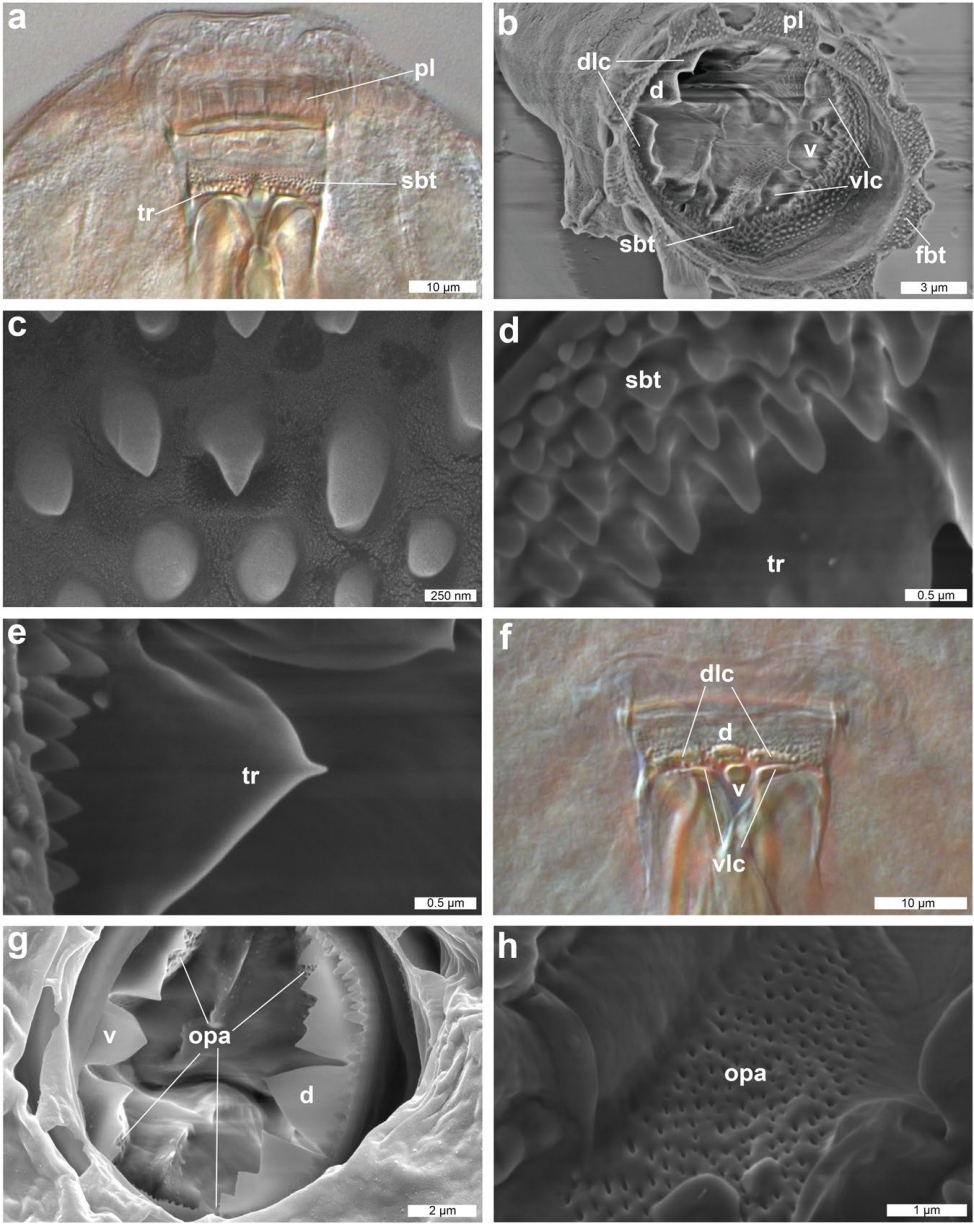
DIC and SEM images of mouth structures of *Dactylobiotus ovimutans* sp. nov. (**a**) The anterior part of the buccal-pharyngeal apparatus (DIC). (**b**) The anterior view of the buccal-pharyngeal apparatus (SEM). (**c**) the first band of teeth (SEM). (**d**) The second band of teeth (SEM). (**e**) the transverse crest (SEM). (**f**) the transverse crests (DIC). (**g**) four oval perforated areas and oral cavity armature (SEM). (**h**) the oval perforated area (SEM). d: dorsomedian transverse crest, dlc: dorsolateral transverse crest, fbt: first band of teeth, opa: oval perforated area, pl: peribuccal lamella, sbt: second band of teeth, tr: transverse crest, v: ventromedian crest, vlc: ventrolateral crest.

**Figure 3:**
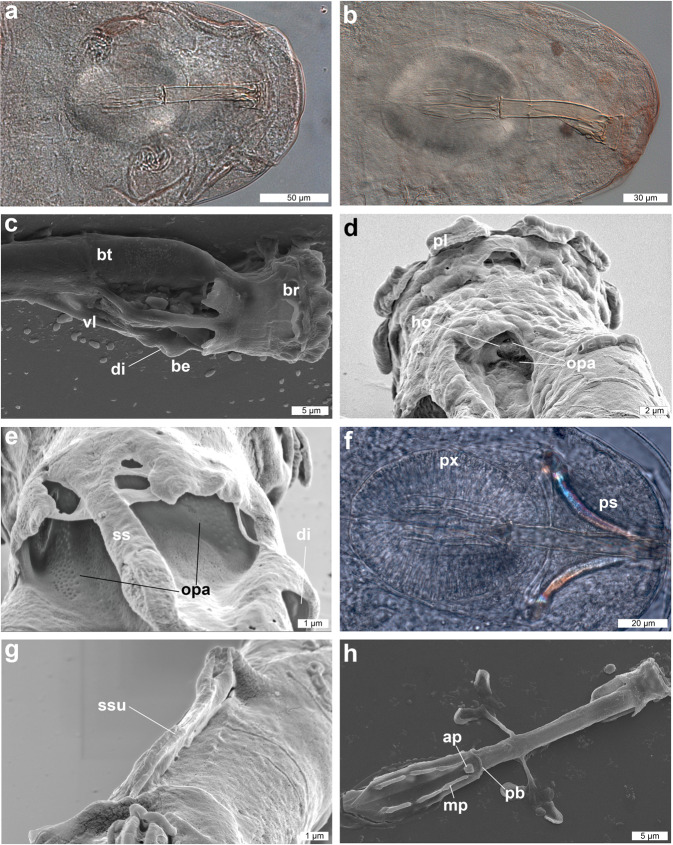
DIC and SEM images of the buccal-pharyngeal apparatus of *Dactylobiotus ovimutans* sp. nov. (**a**) The ventral view of the buccal-pharyngeal apparatus (DIC). (**b**) The lateral view of the buccal-pharyngeal apparatus (DIC). (**c**) The anterior part of the buccal-pharyngeal apparatus (SEM). **(d,e**) The oblique posterior view of the anterior part of the buccal-pharyngeal apparatus (SEM). (**f**) Buccal-pharyngeal apparatus with bended stylets of a live specimen of *Dactylobiotus ovimutans* sp. nov. (DIC). (**g**) Stylet support, which has collapsed back against the buccal tube (SEM). (**h**) Buccal-pharyngeal apparatus (SEM). ap: apophysis, be: bulbous expansion, br: buccal ring, bt: buccal tube, di: deep invagination, ho: hole, mp: macroplacoid, opa: oval perforated area, pb: pharyngeal bar, pl: peribuccal lamella, ps: piercing stylet, px: pharynx, ss: stylet sheath, ssu: stylet support, vl: ventral lamina.

**Figure 4:**
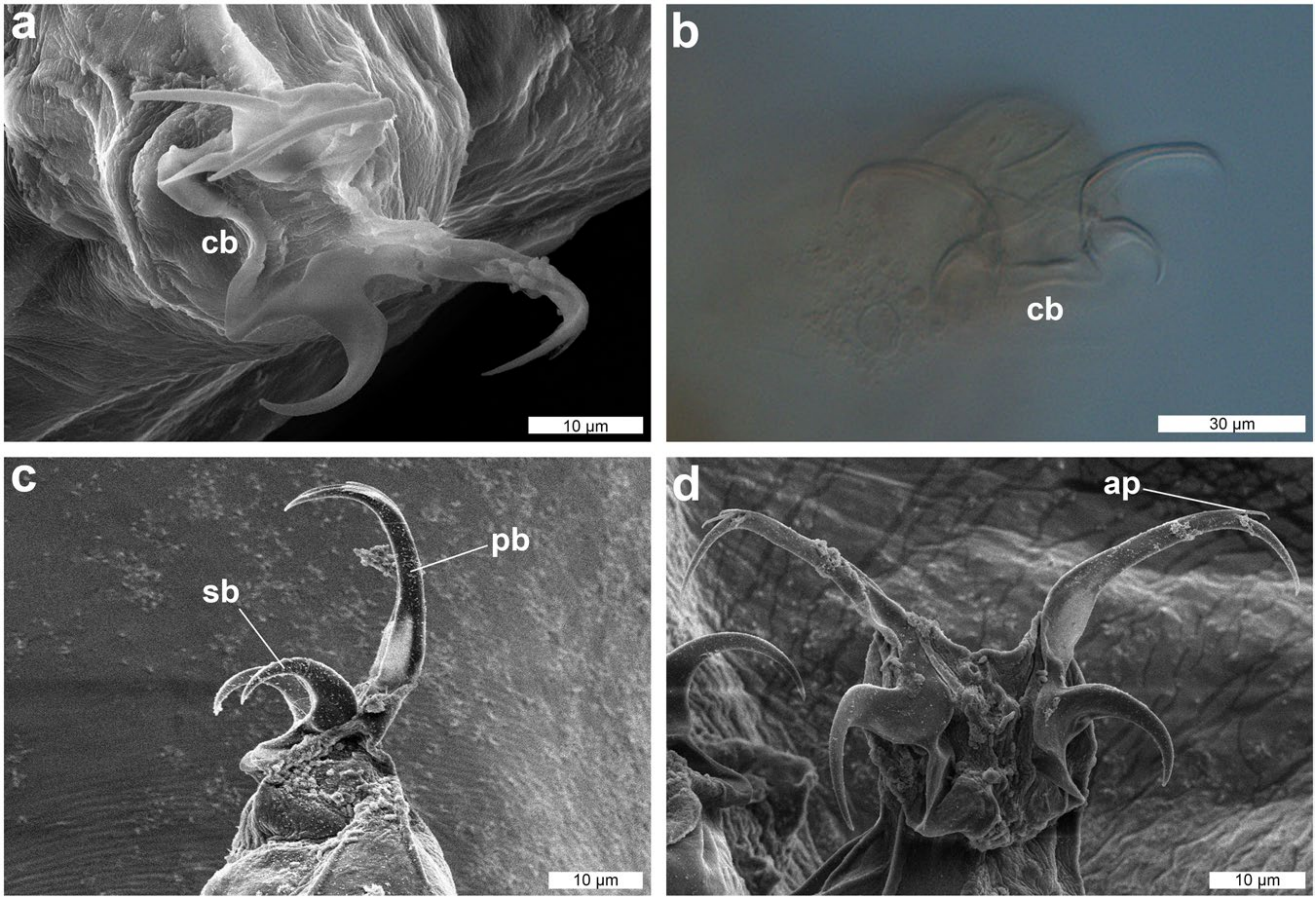
DIC and SEM images of the claw of *Dactylobiotus ovimutans* sp. nov. (**a**) The claw I (SEM). (**b**) The claw II (DIC). (**c**) The claw III (SEM). (**d**) The claw IV (SEM). ap: accessory point, cb: cuticular bar, pb: primary branch, sb: secondary branch.

(Figures 1–4, Supplementary Figure S2; measurement and statistics in Supplementary Table S1)

Body colour white and slightly opaque (in live specimens) or transparent (in Hoyer’s medium). Smooth cuticle without gibbosites, spines and pores. Dorso-lateral papillae also absent, but see remarks below (Fig. 1). Eyes present in live specimens (Fig. 1c), and most specimens show eyes after mounting in Hoyer’s medium. Ten peribuccal lamellae present (Fig. 2a,b) with the hook-shaped first band of teeth at the base (Fig. 2c). The first band of teeth (FBT) faint under Differential Interference Contrast (DIC) microscope, but clearly visible under SEM (Fig. 2a,b). The second band of teeth (SBT) larger caudally (Fig. 2d), followed by dorsal and ventral transverse crests. In most observed specimens, dorsomedian and ventromedian transverse crests with a pointed tip (Fig. 2e,f, Supplementary Fig. S2). The dorsomedian crest slightly broader than the ventromedian crest (Fig. 2f,g). Dorsolateral and ventrolateral crests have similar size and several tips, not fragmented (Fig. 2f,g). Two oval perforated areas (porous areas^56^) on the lateral sides posterior to transverse crests, respectively (Fig. 2g,h). The buccal tube (Fig. 3a), with slight antero-ventral bend (Fig. 3b). The asymmetric apophysis for the insertion of the stylet muscles (AISM) accentuated by the ventral lamina and shows the bulbous expansion with the deep invagination (or hook^53^) (Fig. 3c). At the view from posterior to anterior direction, oval perforated area goes through the hole and into the mouth (Fig. 3d), one dorsal and one ventral to the stylet sheath (Fig. 3e). Two holes and two oval perforated areas in each lateral side of buccal crown, hence four holes and oval perforated areas in total. Piercing stylets of a living specimen 60 μm long with curved shape present (Fig. 3f). Stylet supports (Fig. 3g) inserted on the buccal tube at posterior position. The pharyngeal bar and three bi-lobed pharyngeal apophyses (Fig. 3h) at the posterior end of the buccal tube. Pharynx with two rod-shaped macroplacoids. The first macroplacoid with central constriction at the middle. Microplacoid and septulum absent (Fig. 3a,b,f,h). Claws of *Dactylobiotus* type (2–1–1–2) with a cuticular bar between the claw bases (Fig. 4a,b). While claws on the first three pairs of legs sub-equal in length and proportion, claws on the last pairs clearly longer. Accessory points are present on the primary branches of all claws (Fig. 4c,d). Lunules absent.

**Figure 5:**
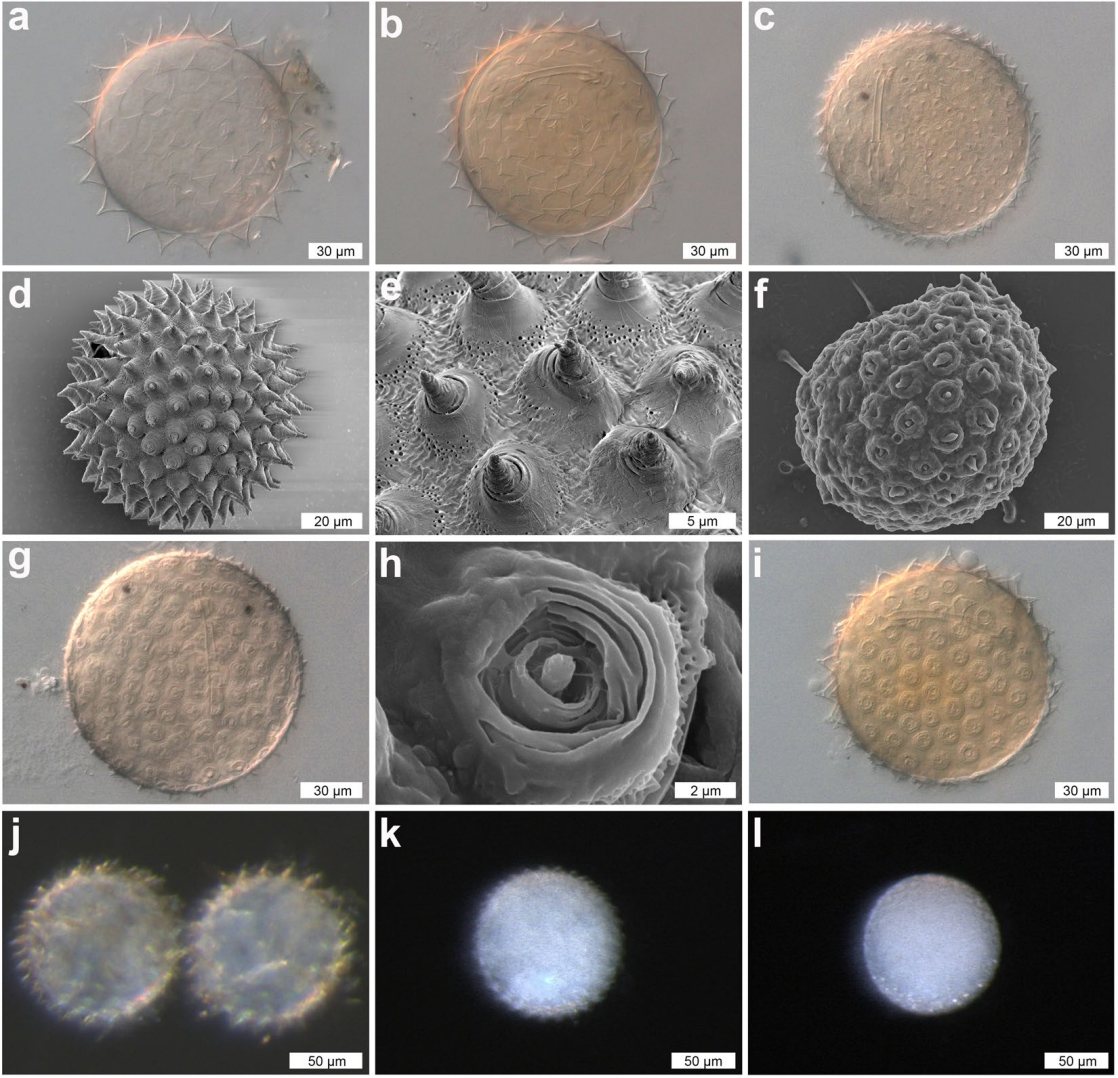
DIC, SEM, and stereoscopic microscope images of the eggs of *Dactylobiotus ovimutans* sp. nov. (**a**) An egg with 20 processes on the circumference (DIC). (**b**) An egg with 25 processes on the circumference (DIC). (**c**) An egg with 37 processes on the circumference (DIC). (**d**) An egg shell (SEM). (**e**) Processes of an egg (SEM). (**f**) An egg with uninflated processes (SEM). (**g**) An egg with uninflated processes (DIC). (**h**) An uninflated process (SEM). (**i**) An egg with partially inflated/uninflated processes (DIC). (**j–l**) Images of the eggs used for DNA extraction. (**j**) Two eggs with 25 and 22 processes on the circumference (stereoscopic). (**k**) An egg with 34 processes on the circumference (stereoscopic). (**l**) An egg with uninflated processes (stereoscopic).

**Eggs**

(Figure 5; Supplementary Tables S2)

Large, white or slightly yellow, laid freely. Spherical or slightly oval. Transparent or slightly yellow after mounting in Hoyer’s medium. Number of processes on the circumference varying from 20 to 37 (Fig. 5a–c). Process in the shape of a cone with circular bases (Fig. 5d), several concentric ring ridges on the surface with a single tip; though some are bi- or multi-furcated (Fig. 5e). Concentric ring ridges on the processes more or less pronounced depending on the level of ‘inflation’ (Fig. 5d–h). An irregular ring of fine pores encircling the processes, causing slight, uneven ridging of the eggshell between the processes (Fig. 5e). All the egg structures mentioned above are visible under both DIC and SEM observations.

**Molecular results**

(Supplementary Table S3)

All sequences of *Dactylobiotus ovimutans* sp. nov. are deposited in GenBank under accession numbers: MT136805 (18S), MT136807 (28S), MT132332 (COI haplotype 1), MT132333 (COI haplotype 2).

In terms of molecular gene sequences, only three named *Dactylobiotus* species are registered at GenBank, with several not identified to species level. The named individuals are: *Dac. parthenogeneticus* Bertolani, 1982^57^, with partial gene sequences of COI (GenBank accession number AY598771)^58^, 18S rRNA (GenBank accession number HQ604963, HQ604964)^59^ and trehalose-6-phophate synthase (TPS) (GenBank accession number JF488058)^60^; *Dac. octavi* with partial sequences of 18S rRNA (GenBank accession number GQ849025)^61^ and 28S rRNA (GenBank accession number GQ849049)^61^; and *Dac. ambiguus* with partial sequence of 18S rRNA (GenBank accession number GQ925676*–*925681)^62^ and 28S rRNA (GenBank accession number MH079500)^63^.

We found the same haplotypes for 18S rRNA (1599 bp) and 28S rRNA (533 bp) in two analysed individuals. There are two haplotypes of two bp differences in the partial sequences of COI (673 bp) of 21 individuals and 19 eggs (Supplementary Figure S3). Seven individuals and four eggs belong to haplotype 1, and fourteen individuals and fifteen eggs belong to haplotype 2. The sequence reported for a *Dactylobiotus* sp. (GenBank accession number EF632526)^64^ (collected from moss at Lake Jubany (ca. 62°14ʹ S; 58°40ʹ W), *c*. 4 Km from Lake CZO), was identical to *Dac. ovimutans* sp. nov. haplotype 1 (Supplementary Figure S3). The analysis of p-distances for COI involved four nucleotide sequences. There was a total of 518-bp positions in the final dataset. The p-distances for COI between *Dac. ovimutans* sp. nov. haplotypes was 0.001%; haplotype 1, haplotype 2, *Dactylobiotus* sp. (GenBank accession number: EF632526, Lake Jubany) and *Dac. parthenogeneticus* (AY598771) varied between 0–16.5%. Between *Dac. ovimutans* sp. nov. and *Dac. parthenogeneticus* (HQ604963) there are 2 bp changes in 738 bp for 18S (see: Supplementary Fig. S4).

The new species and *Dac. octavi* (Disko Island, Greenland) has 4 bp changes in 1599 bp for 18S rRNA (GenBank accession number GQ849025), and 1 bp change in 504 bp for 28S rRNA (GenBank accession number GQ849049). Between *Dac. ovimutans* sp. nov. and *Dac. ambiguus* (Alabama, USA) there are 5 bp changes in 1599 bp for 18S (GenBank accession number GQ925676).

**-Etymology**

From the Latin *ovimutans* ‘egg-changing’ to reflect the variation in egg processes.

Type depositories: Slides of the holotype and paratype are held in the collection at the Division of Polar Life Sciences, KOPRI (Korea Polar Research Institute). Slides of four individuals and an egg (slide labels *Dactylobiotus* Sejong 46, 49, 52, 53, and *Dactylobiotus* Sejong egg 07‒09) are held in the collection at the Department of Animal Taxonomy and Ecology, Faculty of Biology, Adam Mickiewicz University (Poland).

### Remarks

Species which belong to the genus *Dactylobiotus* are characterized in having the same number of macroplacoids, similar shape of AISM and bulbous expansion, and cuticular bars between claws, but differ mostly in the eggshell morphology^38,39,65–69^. Nevertheless, some morphological traits may have been overlooked in previous, particularly early, descriptions. For example, FBT is faint or invisible under the Phase-Contrast microscope (PCM) or DIC microscope, thus the presence or absence of this character in other *Dactylobiotus* species needs to be verified. We also noted that measurements of most of other *Dactylobiotus* species were based on only few specimens, which hinders understanding the variation of taxonomically important traits. However, the morphometric characters available for these species have enable us to define the new species of *Dac. ovimutans* sp. nov.

The presence of dorso-lateral papillae between the third and fourth limbs is an important morphological character that distinguishes three species from others within the genus, i.e. *Dac. dispar*, *Dac. selenicus* Bertolani, 1982^57^ and *Dac. parthenogeneticus*. In the new species, a dorso-lateral papillae-like structure was observed in only one individual SEM specimen among 9 SEM and 61 DIC specimens. We therefore believe the presence of dorso-lateral papillae in *Dac. ovimutans* sp. nov. is doubtful. More individuals with a dorso-lateral papillae-like structure need to be studied in order to reveal whether the structure is an artefact or represents different species.

**-Differential diagnosis**

Seventeen *Dactylobiotus* species have been described^70^, although *Dac. macronyx* Dujardin, 1851^71^ is considered dubious species, which may be a synonym of *Dac. dispar*^66,72^. The validity of the two Chinese species *Dac. aquatilis* Yang, 1999^73^ and *Dac. henanensis* Yang, 2002^74^ requires further study, as taxonomically important characters were not described^68,69^. Thus, we compare our specimens with the descriptions of the fourteen widely accepted species.

Using the most recent keys to the genus *Dactylobiotus*^66^, the new species, *Dactylobiotus ovimutans* sp. nov., is morphologically similar to four species: *Dac. ambiguus*, *Dac. caldarellai*, *Dac. luci* Kaczmarek, Michalczyk and Eggermont, 2008^65^, and *Dac. dervizi* Biserov, 1998^67^. *Dactylobiotus ovimutans* sp. nov. differs specifically from:

***Dactylobiotus ambiguus*** (measurement data^50^), while *Dactylobiotus ambiguus* was originally described from Scotland^38^ without morphometric data, Pilato & Binda^50^ offered morphometric data of *Dac. ambiguus* from Canada and Italy. Hence, in this study the morphometric data of *Dac. ambiguus* from Pilato & Binda^50^ were used. The percent ratio of the length of a character to the length of buccal tube (*pt*) value of claw IV primary branch is lower in the new species (*40.75–72.87 vs*. ca. *82.66* in *Dac. ambiguus*), with a slightly higher percent ratio of the length of the secondary branch to the primary branch (*br)* value of claw IV (*35.14–51.60 vs*. ca. *33.99* in *Dac. ambiguus*). The closely packed egg processes of *Dac. ambiguus* produce a strongly polygonal base, while the larger distance between the processes of *Dac. ovimutans* sp. nov. give a circular to weakly hexagonal base. The outline of the egg processes of *Dac. ovimutans* sp. nov. is more triangular with a relatively large tip, while that of *Dac. ambiguus* was described as oval and acuminate^38^.

***Dactylobiotus caldarellai*** (measurement data^72^), recorded from Punta Arenas, Chile, is similar to the new species in having well-developed oral armature, although it is not clear whether the FBT at the base of the peribuccal lamellae is present^50^. Differences are noted between the *pt* value of stylet support insertion point (SSIP) and a shape of claws: slightly lower *pt* value of SSIP (*67.47–77.03 vs*. ca. *77.82* in *Dac. caldarellai*), a lower *br* value at the claw II (*30.97–46.18 vs*. ca. *51.40* in *Dac. caldarellai*). Ventrolateral crests of *Dac. caldarellai* are fragmented, in contrast to *Dac. ovimutans* sp. nov. To avoid the allometric effect, we compared the morphometry between similar sized specimens of the two species, and the disparity become more pronounced. Although the buccal tube lengths in the two specimens used for comparison were similar (54.24 μm *vs*. 52.20 μm in *Dac. caldarellai*), *Dac. ovimutans* sp. nov. shows a higher ventral lamina *pt* value (*56.90 vs. 43.00* in *Dac. caldarellai*), a lower SSIP *pt* value (*69.32 vs. 77.82* in *Dac. caldarellai*), a higher *pt* value of primary branch of claw II (*48.95 vs. 34.77 in Dac. caldarellai*), a lower *br* value at the claw II (*42.34 vs*. *51.53* in *Dac. caldarellai*), a higher *pt* value of the primary branch of claw IV (*65.69 vs. 44.71* in *Dac. caldarellai*), and a lower *br* value at the claw IV (*37.19 vs. 48.97* in *Dac. caldarellai*) (see: Supplementary Table S4).

***Dactylobiotus luci*** (measurement data^65^), reported from Uganda, is similar to the new species in having well-developed oral cavity armature, but apparently lacks the FBT at the base of the peribuccal lamellae. The eggs of the new species have broader process base width (10.54–21.00 µm *vs*. 5.2–7.1 µm in *Dac. luci*).

***Dactylobiotus dervizi*** **(**measurement data^67^), recorded from the Komandorskiye Islands, Russia, is similar to the new species in having well-developed oral armature, but apparently without the FBT (Figures 7 & 8^67^). Eyes are absent in *Dac. dervizi* while they are present in the new species. The eggs of the new species have broader process base width (10.54–21.00 µm *vs*. ca. 9.0 µm in *Dac. dervizi*). Eggshell has a discrete ring of pores around the process in the new species and random pores between the processes in *Dac. dervizi*.

## Discussion

### Intraspecific variation in the egg morphology and its implications

During the culturing of *Dactylobiotus ovimutans* sp. nov., we noticed a significant amount of morphological variation in the eggs, which were easily recognized in the number of processes around the circumference of the egg. The variable egg morphology is independent of the two different COI haplotypes (Supplementary Fig. S5). While there was no significant difference in egg diameter, the egg processes differed in size and morphology. We found that eggs with fewer processes had triangular processes with concave wall (Fig. 5a), and eggs with more numerous processes had semi-triangular to hemispheric processes with convex wall (Fig. 5b,c). As the number of egg processes increased, the size of the processes become smaller. There were also some embryonated eggs in a pre-deposition state, i.e. with the processes still uninflated and flattened, or partially inflated (Fig. 5f–i). The causes preventing inflation of the processes was not clear. In an experiment with *Dactylobiotus* sp. from Signy Island lakes to explore desiccation, McInnes *et al*.^75^ did observe collapsed processes under desiccation but flattened side to side, not concertinaed as in the pre-deposition state.

Such strong differences in eggshell morphology were also documented in many limno-terrestrial species. Some species show various convexity on the processes (e.g. refs. ^76,77^), while other species show various process shapes (e.g. refs. ^40,43,78–81^) or process density (e.g. ref. ^82^).

The cause of the difference in eggshell morphology remains elusive. It has been suggested that the dormant and active eggs may show disparate size and morphology (e.g. refs. ^83–85^). However, the variable egg types of *Dac. ovimutans* sp. nov. developed and hatched under the same laboratory condition, so this possibility seems unlikely. Alternatively, different eggs could be formed by seasonality. It has been shown in *Bertolanius nebulosus* (Dastych, 1983)^86^ that through the year long life of an adult, eggs produced in winter had a more enlarged tips of the processes than those produced in summer^41^. However, *Dac. ovimutans* sp. nov. showed that the variable types of egg were laid under the same, stable laboratory condition (temperature, light and food), excluding the possibility of seasonality. For now, the only possibility that cannot be ruled out is a differential gene expression (epigenetic effect), as proposed by Stec *et al*.^42^.

### The eggs of *Dac. ovimutans* sp. nov. from other studies

Pilato and Binda^50^ suggested that *Dac. caldarellai*, which they described from Chile, was conspecific with the *Dac. ambiguus* described by Dastych^34^ from KGI, and therefore the eggs would be the same. However, while *Dac. caldarellai* has fragmented ventrolateral crests^50^, Dastych’s *Dac. ambiguus* from KGI has concatenate ventrolateral crests^34^. In this study, we have observed that the adult morphology of *Dac. ovimutans* sp. nov. is distinct from both *Dac. caldarellai* and the specimen from Canada which Pilato & Binda^50^ considered to be *Dac. ambiguus* (original description of *Dac. ambiguus*^38^ is insufficient for comparison). Dastych provided measurements for a single adult *Dac. ambiguus* reported from KGI^34^, which falls within the measurement range for *Dac. ovimutans* sp. nov., but the data is too limited to confirm the species. Both *Dac. ovimutans* sp. nov. and Dastych’s KGI *Dac. ambiguus*^34^ have concatenate ventrolateral crests, and the second band of teeth decreasing in size towards mouth opening. However, FBT was not observed in Dastych’s *Dac. ambiguus*^34^. The egg morphology of Dastych’s KGI *Dac. ambiguus*^34^ is comparable to that of *Dac. ovimutans* sp. nov., in having similar size, space between processes and an irregular ring of fine pores encircling the processes (narrow wreath of minute spots around the bases of processes, as described by Dastych^34^). In contrast, the egg morphology of the original *Dac. ambiguus*^38^ differs because the processes are packed without space. These facts imply that the eggs from Dastych’s KGI samples^34^ might belong to neither *Dac. caldarellai* nor *Dac. ambiguus,* but to *Dac. ovimutans* sp. nov.

McInnes^47^ recognized two types of eggs of *Dac. ambiguus* from Signy Island, Antarctica and noted that the first type had many processes in which the process bases were connected, while the second type had fewer unconnected processes. The overall morphology of the second type of eggs is reminiscent of the eggs of *Dac. ovimutans* sp. nov., such as egg size, processes with several concentric ring ridges on the surfaces, space between the processes and fine pore rings encircling the processes (see: Supplementary Fig. S6, Supplementary Table S5). Therefore, the second type eggs of *Dac. ambiguus* from Signy Island could in fact be the eggs of *Dac. ovimutans* sp. nov. Taking into account high dispersal abilities of tardigrades by wind^87–89^ as well as birds^35,90^, wide dispersal of resting forms being transported between Antarctic islands is plausible. Hence, the similarity of morphology of egg type described for Signy Island *Dac. ambiguus* indicates that these specimens should be considered as *Dactylobiotus* cf. *ovimutans* sp. nov, but further analysis is required for confirmation.

### Diet of *Dac. ovimutans* sp. nov

Unlike other cultures of *Dactylobiotus* species that were given a herbivorous diet (e.g. *Dac. dispar*^91^ and *Dac. parthenogeneticus*^92^), *Dac. ovimutans* sp. nov. fed on rotifers from the Lake CZO. Under the culture feeding on rotifers only, *Dac. ovimutans* sp. nov. were vigorous and laid many eggs with varying morphology. We recorded *Dac. ovimutans* sp. nov. feeding on rotifers (Supplementary Movie S1) and noted that after ingesting rotifers the gut of *Dac. ovimutans* sp. nov. turned a distinctive shade of yellow. However, in the absence of rotifers and cultured on sediments containing organic material from lake CZO, the gut of *Dac. ovimutans* sp. nov. turned green or brown, suggesting ingestion of algae or cyanobacteria. *Dactylobiotus* feeding on a nematode *Plectus* sp. was observed from a culture of Signy Island lake sediments with a mix of native species (McInnes pers. obs.). Therefore, we conclude that *Dac. ovimutans* sp. nov. is omnivorous.

### Summary

A new species *Dactylobiotus ovimutans* from King George Island, Antarctica is distinguished from *Dac. ambiguus* and *Dac. caldarellai* in having different claw morphology. The presence of *Dac. ambiguus* and *Dac. caldarellai* on KGI and other regions of the maritime Antarctic should be considered dubious, and/or requires further detailed, integrated taxonomic analysis. Culturing of *Dac. ovimutans* sp. nov. revealed a significant variation in the eggshell morphology, which was recognized by differences in the number and size of the processes. The eggs with fewer processes had larger and concave process profiles, while those with more numerous processes were smaller and convex process profile. Some eggs retained uninflated processes as in the pre-deposition state. Since all eggs were laid in the same stable laboratory condition, such variation is considered to be caused by epigenetic effects, and not being subject to different temperature, food source and seasonality.

## Material and Methods

### Sample & Specimens

The KOPRI (Korea Polar Research Institute) ecology team near the King Sejong Station, KGI, Antarctica (62°14′24.140″S, 58°44′36.571″W), collected benthic sediment samples from the Lake CZO. The sediment contained organic material, i.e. algae and moss. Samples were brought to KOPRI (Incheon, Korea), stored in 4 °C for several months, and eight tardigrades were extracted using a stereomicroscope (Leica M205C). Individuals were cultured separately at 11 °C on a 1.5% bacto-agar plate with Volvic^®^ water, with food source provided as rotifers and algae collected from the King George Island moss samples.

For light microscopic observation, tardigrades were prepared using thermal relaxation by incubating live individuals at 60 °C for 30 min (following ref. ^93^), and mounted on Higgins-Shirayama slide (HS-slide)^94^ in Hoyer’s medium. After drying seven days at 60 °C, the slides were sealed with nail polish, and examined under a Differential Interference Contrast (DIC) microscope (Carl Zeiss Axio Imager 2), with the camera AxioCam HRc. Additional tardigrades were prepared for SEM using thermal relaxation and the method in ref. ^95^. SEM observations were made using a JEOL JSM-6610 and Field Emission SEM JSM-7200F, at KOPRI for KGI specimens. SEM observations of eggs from Signy Island have been conducted in British Antarctic Survey (BAS) following ref. ^75^.

Twenty-one adult tardigrades and nineteen eggs, from the cultured animals, were selected randomly and the identities were checked under a stereo microscope. Then we extracted DNA from these individuals and eggs using commercial kits (TIANamp Micro DNA Kit and QIAamp DNA Micro Kit).

For classical taxonomic observations, we examined 61 individuals and 59 egg specimens under DIC, and 9 adult and 14 egg specimens under SEM. All specimens from this study were registered with ATNS (Antarctic Tardigrade Name of Specimen) numbers and stored in KOPRI.

### Morphometrics

Character selection and measurements followed Binda & Pilato^72^. All measurements are in micrometres (μm). Characters were measured when the specimens were presented in the correct orientation on the slide. Body length was measured from the anterior tip to the end of the body, excluding the leg IV, and *pt* index is the percent ratio of the length of a character to the length of buccal tube^96^. In the claw measurements, the *br* index is the percent ratio of the length of the secondary branch to the length of the primary branch^56^. Allometric measurements were calculated with the exponent (b) and the Y-intercept (a*) of the regression of Thorpe’s normalized characters versus body size^97^. Most of the morphological terminology follows Ramazzotti & Maucci^98^. However, several characters have been more recent revisions^53,99,100^, i.e. ‘the first band of teeth’, ‘the second band of teeth’, ‘oval perforated area’, ‘apophysis for the insertion of the stylet muscles’, and the claw’s ‘cuticular bar’.

### Molecular data

DNA was extracted from two individuals using TIANamp Micro DNA kit, and nineteen individuals and nineteen eggs using QIAamp DNA Micro Kit. We acquired three DNA partial genes from two individuals: 18S rRNA, 28S rRNA, and COI, and acquired COI partial gene sequences from nineteen individuals and nineteen eggs. The PCR mixture was prepared in a total volume 50 μl with Takara EmeraldAmp® GT PCR Master Mix 25 μl, 0.5 μl of each primer, 2 μl of DNA template and 22 μl of *dd*H_2_O. The primers and PCR programmes are in Supplementary Table S6 & S7. PCR products were sent to the commercial company for sequencing (Macrogen, Korea). To calculate molecular distances for the COI fragments, two sequences were obtained from GenBank for *Dactylobiotus* (GenBank accession number EF632526^64^, AY598771^58^). Sequences were processed in BioEdit ver.7.2.5^101^. Pairwise distances between nucleotide sequences were calculated using a distance model for all codon positions as implemented in MEGA X^102^. p-distance calculations for all positions containing gaps and missing data were eliminated.

### Nomenclatural acts

This published work and the nomenclatural acts it contains have been registered in ZooBank, the online registration system for the International Code of Zoological Nomenclature (ICZN). The ZooBank LSIDs (Life Science Identifiers) can be resolved and the associated information viewed through any standard web browser by appending the LSID to the prefix ‘http://zoobank.org/’. The LSID for this publication is: urn:lsid:zoobank.org:pub:362EE4A0‒A6F2‒4F78‒AF65‒EA87028EA1DD.

## Supplementary information

Supplementary Movie S1 Online.

Supplementary information.

## Data Availability

The datasets generated during and/or analysed during the current study are available from the corresponding author on reasonable request.
