# Supplementary material for: Integrative description of a new Dactylobiotus (Eutardigrada: Parachela) from Antarctica that reveals an intraspecific variation in tardigrade egg morphology
Source: Sci Rep. 2020 Jun 4;10:9122. doi: 10.1038/s41598-020-65573-1 (PMC7272612; doi:10.1038/s41598-020-65573-1)
Supplement: Supplementary file 2 — Supplementary information. [file 41598_2020_65573_MOESM2_ESM.pdf]

Integrative description of A new *Dactylobiotus* (Eutardigrada: Parachela) from Antarctica that reveals an intraspecific variation in tardigrade egg morphology

Ji-Hoon Kihm[1,2], Sanghee Kim[3], Sandra J. McInnes[4], Krzysztof Zawierucha[5], Hyun Soo Rho[6], Pilmo Kang[1], Tae-Yoon S. Park[1,*]

[1] *Division of Polar Earth-System Sciences, Korea Polar Research Institute, 26 Songdomirae-ro, Yeonsu-gu, 21990 Incheon, Korea*

[2] *Polar Science, University of Science & Technology, 217 Gajeong-ro, Yuseong-gu, 34113 Daejeon, Korea*

[3] *Division of Polar Life Sciences, Korea Polar Research Institute, 26 Songdomirae-ro, Yeonsu-gu, 21990 Incheon, Korea*

[4] *British Antarctic Survey, Natural Environment Research Council, High Cross, Madingley Road, Cambridge, CB3 0ET, UK*

[5] *Department of Animal Taxonomy and Ecology, Faculty of Biology, Adam Mickiewicz University, Poznań, Uniwersytetu Poznańskiego 6, 61-614 Poznań, Poland*

[6] *East Sea Environment Research Center, East Sea Research Institute, Korea Institute of Ocean Science & Technology, 48 Haeyanggwahak-gil, Uljin, 36315 Gyeongsangbuk-do, Korea*

* Corresponding author: typark@kopri.re.kr

Supplementary Figures and Tables:

Supplementary Figure S1

Study area. a, Antarctica (square: King George Island, arrow: Signy Island). b, King George Island, showing Lake CZO and Lake Jubany of Barton Peninsula and Potter Peninsula respectively.

Supplementary Figure S2

The transverse crests of *Dactylobiotus ovimutans* sp. nov. a, The mouth opening which shows the dorso and the ventromedian transverse crest with pointed tips. b, The mouth opening which shows a pointed tip on the dorsomedian transverse crest only. c, The mouth opening which shows a pointed tip on the dorsomedian transverse crest only. The dorsomedian crest and dorsolateral transverse crest combine. d, The mouth opening which shows a pointed tip on the ventromedian transverse crest only. d: dorsomedian transverse crest, dlc: dorsolateral transverse crest, opa: oval perforated area, v: ventromedian transverse crest, vlc: ventrolateral transverse crest.

Supplementary Figure S3

The cytochrome c oxidase subunit I (COI) alignment of *Dactylobiotus ovimutans* sp. nov. haplotype 1 and 2. A site highlighted in green indicates substitution between haplotype1-*Dactylobiotus* sp. (EF632526) and haplotype 2, yellow indicates substitutions between haplotype 1 and 2.

Supplementary Figure S4

The small ribosome subunit (18S rRNA) alignment of *Dactylobiotus ovimutans* sp. nov. and *D. parthenogeneticus* (HQ604963). Sites highlighted in yellow indicate substitutions.

Supplementary Figure S5

Eggs that DNA extracted in this study. Number indicates the number of processes on the circumference and number in parentheses indicates haplotype 1 or 2.

Supplementary Figure S6

Phase-contrast microscope (PCM) and Scanning Electron Microscope (SEM) images of eggs of *Dactylobiotus* cf. *ovimutans* sp. nov. from Signy Island, Antarctica. a–c, PCM images of eggs with various numbers of processes. d–g, PCM images of processes of eggs. h–i, SEM image of an egg.

Supplementary Table S1

Measurements (in μm) of selected morphological characters of *Dactylobiotus ovimutans* sp. nov. N, the number of specimens; *pt*, the percent ratio of the length of a character to the length of buccal tube; SD, standard deviation; b, allometric exponent; a*, Y-intercept of the regression line of the Thorpe's normalized trait versus buccal tube length; *br*, the percent ratio of the length of the secondary branch to the length of the primary branch of claw.

Supplementary Table S2

Measurements (in μm) of selected morphological characters of eggs of *Dactylobiotus ovimutans* sp. nov. N, the number of specimens; SD, standard deviation.

Supplementary Table S3

Partial gene sequences of cytochrome c oxidase subunit I (COI), small ribosome subunit (18S rRNA), and large ribosome subunit (28S rRNA) of *Dactyolobiotus ovimutans* sp. nov.

Supplementary Table S4

Morphometric comparison between *Dactylobiotus ovimutans* sp. nov. and *D. caldarellai. pt*, the percent ratio of the length of a character to the length of buccal tube; *br*, the percent ratio of the length of the secondary branch to the length of the primary branch of claw.

Supplementary Table S5

Measurements of *Dactylobiotus* cf. *ovimutans* sp. nov. eggs from Signy Island. All eggs were isolated from the same freshwater sample.

Supplementary Table S6

Primers and references for PCR of a cytochrome c oxidase subunit I (COI), a small ribosomal subunit (18S rRNA) and a large ribosomal subunit (28S rRNA) in this study.

Supplementary Table S7

PCR programmes and references for cytochrome c oxidase subunit I (COI), small ribosomal subunit (18S rRNA) and large ribosomal subunit (28S rRNA) in this study.

Supplementary Movie S1

Movie that *Dactylobiotus ovimutans* sp. nov. feeds on a rotifer.

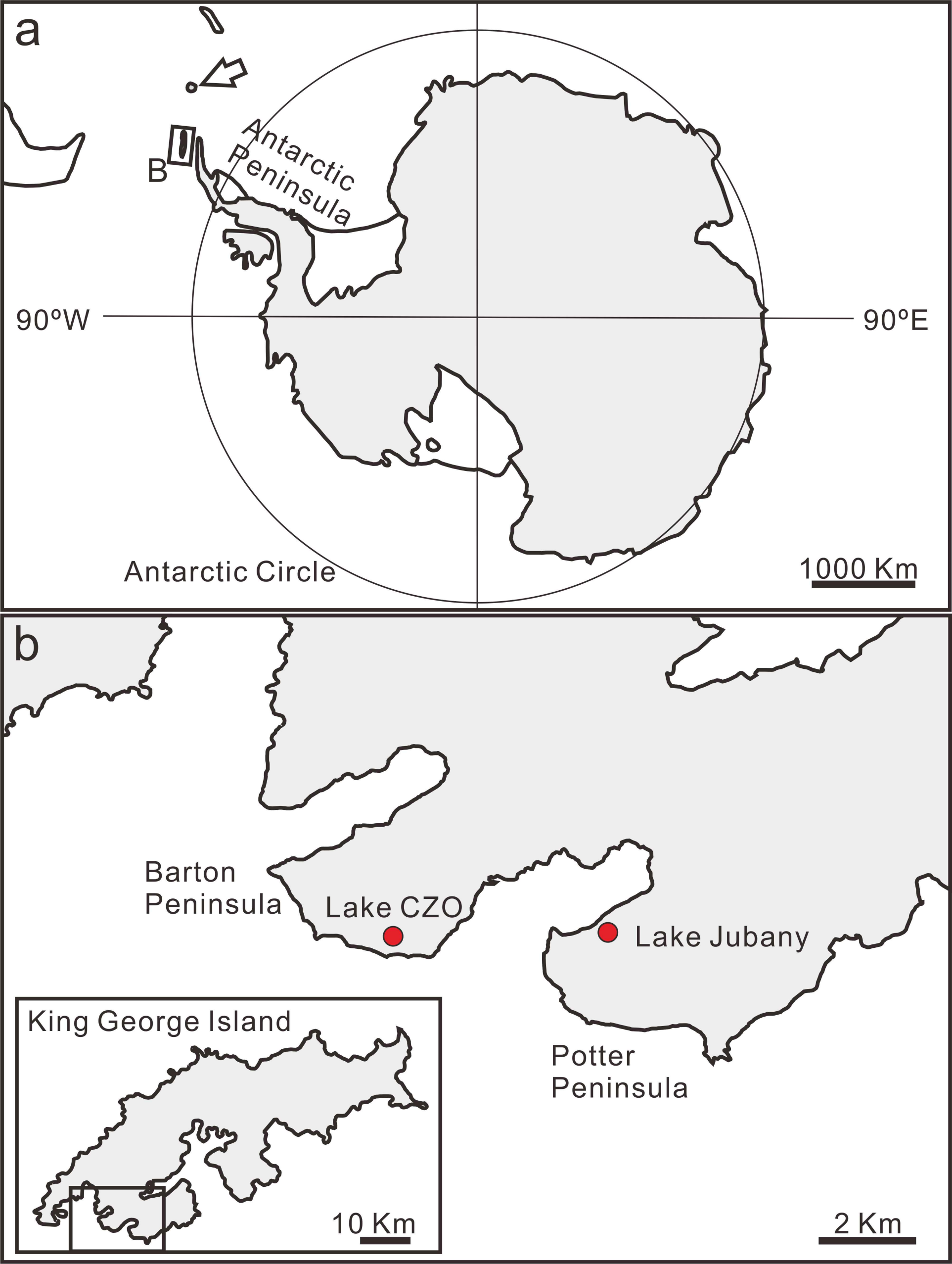

a
Antarctic Peninsula
B
90ºW
90ºE
Antarctic Circle
1000 Km
b
Barton Peninsula
Lake CZO
Lake Jubany
Potter Peninsula
King George Island
10 Km
2 Km

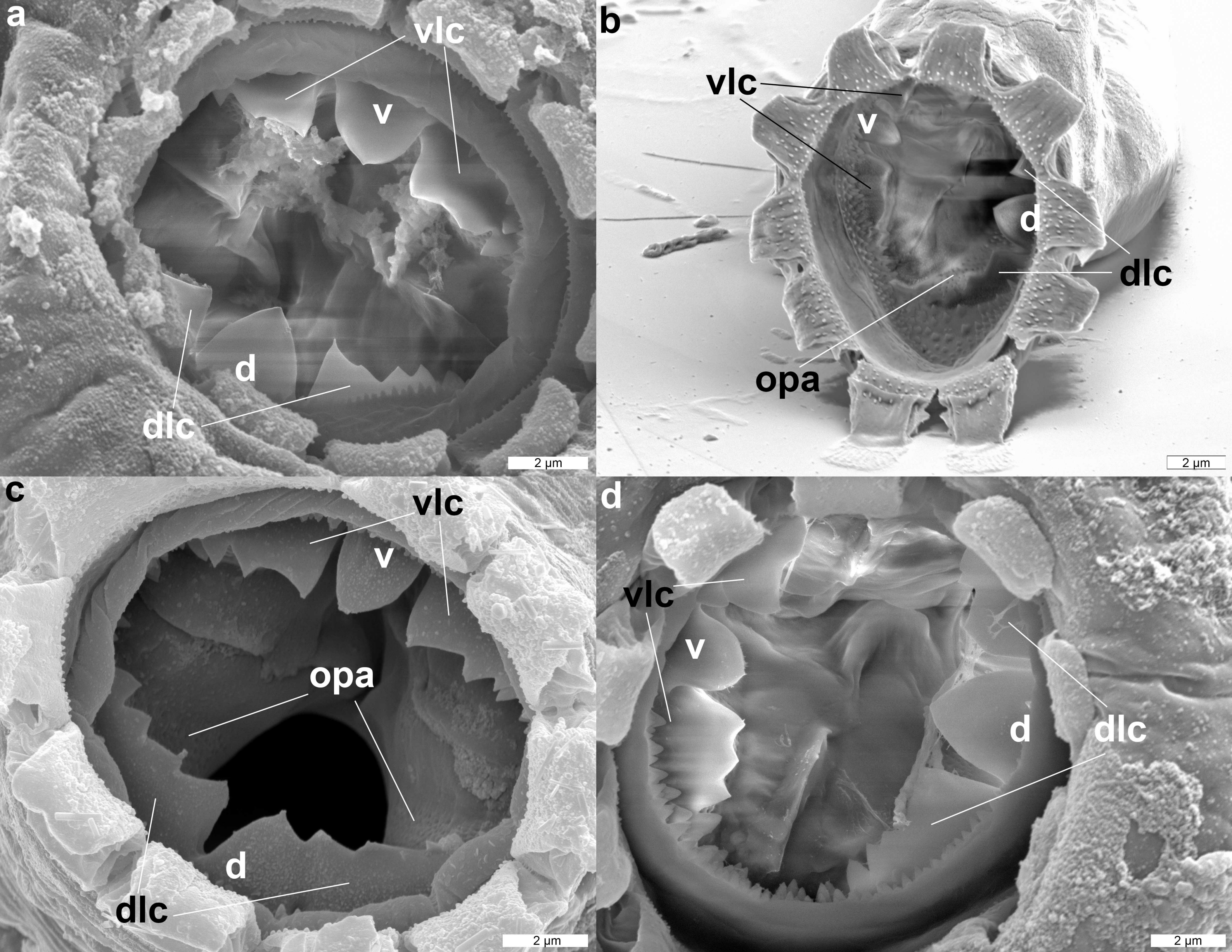
a
vlc
v
d
dlc
2 µm
b
vlc
v
d
dlc
opa
2 µm
c
vlc
v
opa
d
dlc
2 µm
d
vlc
v
d
dlc
2 µm

Haplotype 1 5'-ATATTGGAACACTATATTTTATTTTCGGCCTATGGGCTGCTACCGTAGGAACTTCACTAAGATTTATTATTCGATCTGAATTAAGACAACCTGGCTCTATTTTAATA

Haplotype 2 5'-ATATTGGAACACTATATTTTATTTTCGGCCTATGGGCTGCTACCGTAGGAACTTCACTAAGATTTATTATTCGATCTGAATTAAGACAACCTGGCTCTATTTTAATA

Haplotype 1 GACGATCAAATATATAATGTAATTGTAACTAGACATGCATTTATTATAATTTTCTTTTTCGTAATACCTATTCTTATTGGGGGATTTGGAAACTGACTAGTGCCCTTA

Haplotype 2 GACGATCAAATATATAATGTAATTGTAACTAGACATGCATTTATTATAATTTTCTTTTTCGTAATACCTATTCTTATTGGGGGATTTGGAAACTGACTAGTGCCCTTA

Haplotype 1 ATAATCGGAGCCCCAGATATAGCTTTCCCACGAATAAATAATTTAAGATTCTGACTGCTACCCCCCTCATTCATACTAATTACCAGAGGTACAATAAGAGAACAA

Haplotype 2 ATAATCGGAGCCCCAGATATGGCTTTCCCACGAATAAATAATTTAAGATTCTGACTGCTACCCCCCTCATTCATACTAATTACCAGAGGTACAATAAGAGAACAA

Haplotype 1 GGAGCTGGAACAGGATGAACAGTTTACCCGCCTTTATCCCAATACTTCGCCCATAGTGGACCAAGTGTAGACCTAACTATTTTCTCCCTACATATTGCAGGAGT

Haplotype 2 GGAGCTGGAACAGGATGAACAGTTTACCCGCCTTTATCCCAATACTTCGCCCATAGTGGACCAAGTGTAGACCTAACTATTTTCTCCCTACATATTGCAGGAGT

Haplotype 1 ATCATCCATTCTGGGTGCAATTAATTTTATCTCCACTATTATTAATATACGATCACCAGCAATAAGAATAGAAAATATGCCGCTATTCGTGTGATCTGTACTAATTAC

Haplotype 2 ATCATCCATTCTGGGTGCAATTAATTTTATCTCCACTATTATTAATATACGATCACCAGCAATAAGAATAGAAAATATGCCGCTATTCGTGTGATCTGTACTAATTAC

Haplotype 1 AGCAATCTTACTACTACTTGCCCTACCTGTACTAGCTGGGGGTATTACAATACTACTACTAGATCGTAATTTTAATACTTCATTCTTTGATCCTGCAGGAGGGGG

Haplotype 2 AGCAATCTTACTACTACTTGCCCTACCTGTACTAGCTGGGGGTATTACAATACTACTACTAGATCGTAATTTTAATACTTCATTCTTTGATCCTGCAGGGGGGGG

Haplotype 1 AGATCCAATTCTATACCAACACTTATTCTGATTTTT-3'

Haplotype 2 AGATCCAATTCTATACCAACACTTATTCTGATTTTT-3'

Dac. ovimutans	5'-- TTCTAGAGCTAATACGTGCAATCAGCTTGCTCTCTCGGGAGCAAGCG
Dac. parthenogeneticus	5'-TAAATCAGTTATGGTTCACTAGATCGTACAGTTTACACGGATAACTGTGGTAATTCTAGAGCTAATACGTGCAATCAGCTTGCTCTCTCGGGAGCAAGCG
Dac. ovimutans	CAGTTATTAGAATAAAAACCAATCCGGCCCTCGGGTCGGTACAATTGGTGACTCTGAATAACCGAAGCGGAGCGCATGGTCTCGTACCGGCGCCAGATCT
Dac. parthenogeneticus	CAGTTATTAGAATAAAAACCAATCCGGCCCTTGGGTCGGTACAATTGGTGACTCTGAATAACCGAAGCGGAGCGCATGGTCTCGTACCGGCGCCAGATCT
Dac. ovimutans	TTCAAGTGTCTGACTTATCAGCTTGTTGTTAGGTTATGTTCCTAACAAGGCTTCAACGGGTAACGGGGTATCAGGGTCCGATACCGGAGAGGGAGCCTGA
Dac. parthenogeneticus	TTCAAGTGTCTGACTTATCAGCTTGTTGTTAGGTTATGTTCCTAACAAGGCTTCAACGGGTAACGGGGTATCAGGGTCCGATACCGGAGAGGGAGCCTGA
Dac. ovimutans	GAAACGGCTACCACATCCAAGGAAGGCAGCAGGCGCGCAAATTACCCACTCCCGGCACGGGGAGGTAGTGACGAAAAATAACGATGCGAGAGCTCTAAGC
Dac. parthenogeneticus	GAAACGGCTACCACATCCAAGGAAGGCAGCAGGCGCGCAAATTACCCACTCCCGGCACGGGGAGGTAGTGACGAAAAATAACGATGCGAGAGCTCTAAGC
Dac. ovimutans	TCCTCGTAATCGGAATGGGTACACTTTAAATCCTTTAACGAGGATCTATTGGAGGGCAAGTCTGGTGCCAGCAGCCGCGGTAATTCCAGCTCCAATAGCG
Dac. parthenogeneticus	TCCTCGTAATCGGAATGGGTACACTTTAAATCCTTTAACGAGGATCTATTGGAGGGCAAGTCTGGTGCCAGCAGCCGCGGTAATTCCAGCTCCAATAGCG
Dac. ovimutans	TATATTAAAGTTGCTGCGGTTAAAAAGCTCGTAGTTGGATCTGGGTAGCTGGATGGATGGTGTGCCTTGCGGTATTACTGTCTGTTCGGCACCACAAGCC
Dac. parthenogeneticus	TATATTAAAGTTGCTGCGGTTAAAAAGCTCGTAGTTGGATCTGGGTAGCTGGATGGATGGTGTGCCTTGCGGTATTACTGTCTGTTCGGCACCACAAGCC
Dac. ovimutans	GGCCATGTCTTGTATGCCCTTCACTGGGTGTACTTGGCGACCGGAACGTTTACTTTGAAAAAATTAGAGTGCTCAAAGCAGGCGTACGGCCTTGCATAAT
Dac. parthenogeneticus	GGCCATGTCTTGTATGCCCTTCACTGGGTGTACTTGGCGACCGGAACGTTTACTTTGAAAAAATTAGAGTGCTCAAAGCAGGCGTACGGCCTTGCATAAT
Dac. ovimutans	GGTGCATGGAATAATGGAATAGGATCTCGGATTCTTTTTTGTTGGTTTTCGGAACTCGAGGTAATGATTAATAGGAACAGACGGGGGCATTCGTATTGCG-3'
Dac. parthenogeneticus	GGTGCATGGAATAATGGAATAGGCTCTCGGATTCTTTTTTGTTGGTTTTCGGAACTCGAGGTAATGATTAATAGGAACAGACGGGGGCATT----------3'

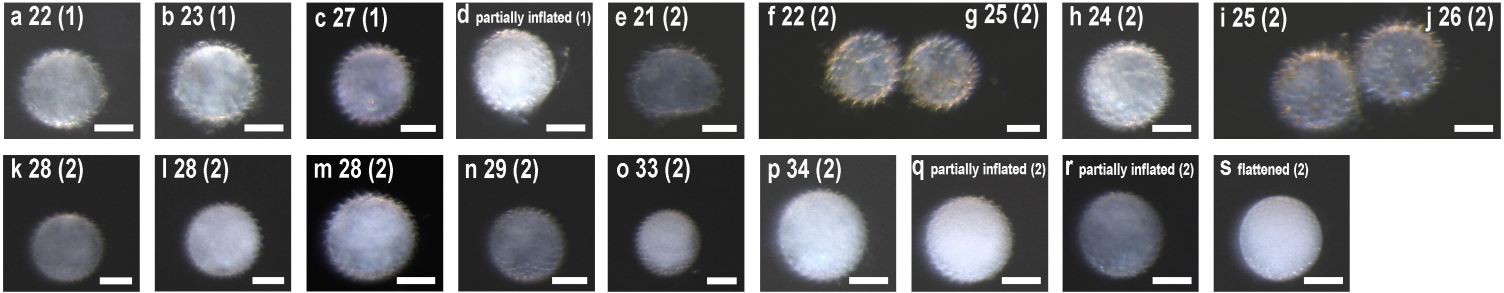
a 22 (1)
b 23 (1)
c 27 (1)
d partially inflated (1)
e 21 (2)
f 22 (2)
g 25 (2)
h 24 (2)
i 25 (2)
j 26 (2)
k 28 (2)
l 28 (2)
m 28 (2)
n 29 (2)
o 33 (2)
p 34 (2)
q partially inflated (2)
r partially inflated (2)
s flattened (2)

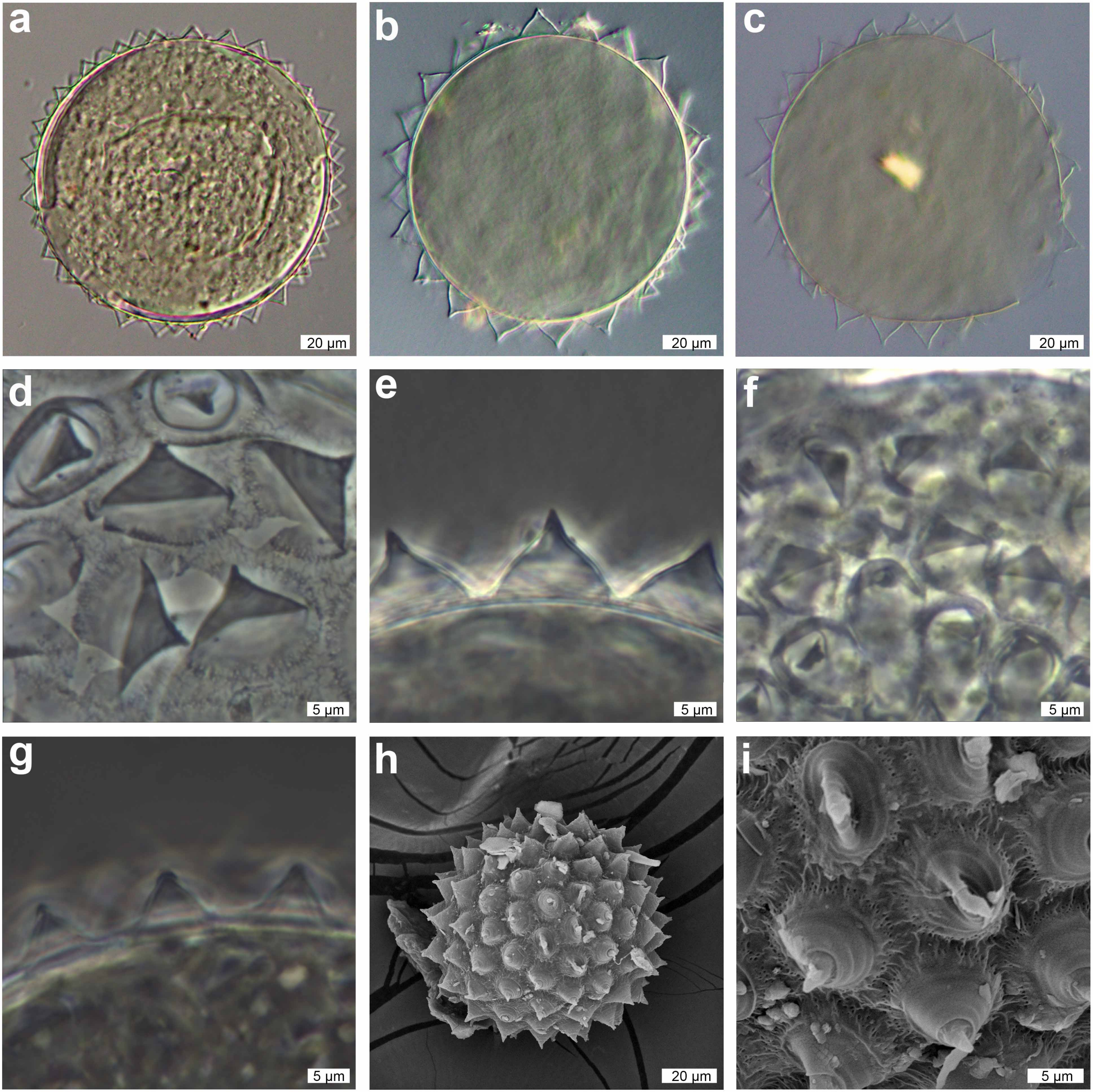
a
20 µm
b
20 µm
c
20 µm
d
5 µm
e
5 µm
f
5 µm
g
5 µm
h
20 µm
i
5 µm

character	N	Range		Average		SD		Holotype		b	a*
		µm	*pt*	µm	*pt*	µm	*pt*	µm	*pt*		
Body length	58	303.49-759.99	*682.44-1123.44*	555.33	*854.39*	127.32	*109.43*	576.32	*868.87*	-	-
**Buccal-pharyngeal tube**											
Buccal tube length	44	37.42-78.02	-	62.84	-	13.05	-	66.33	-	-	-
Stylet support insertion point	44	27.16-57.23	*67.47-77.03*	45.56	*72.65*	9.23	*2.10*	48.93	*73.77*	0.96	45.63
Buccal tube external width	44	4.15-14.01	*11.09-19.31*	9.59	*14.93*	2.98	*2.54*	12.71	*19.16*	1.49	9.43
Buccal tube internal width	44	2.83-11.43	*7.22-15.38*	7.08	*10.96*	2.48	*2.37*	9.99	*15.06*	1.64	6.82
Ventral lamina	41	13.06-42.69	*29.52-67.12*	31.75	*50.30*	7.66	*6.36*	34.08	*51.38*	1.09	32.60
**Placoid lengths**											
Macroplacoid 1	43	10.37-39.69	*27.24-55.03*	26.04	*40.33*	8.20	*6.74*	28.55	*43.04*	1.59	25.74
Macroplacoid 2	43	5.49-21.35	*12.94-28.76*	13.88	*21.44*	4.54	*3.89*	17.40	*26.23*	1.64	13.65
Macroplacoid row	40	17.26-63.56	*42.72-88.58*	42.83	*66.80*	13.98	*11.19*	48.15	*72.59*	1.62	42.89
**Claw I lengths**											
Primary branch	44	13.17-40.37	*35.20-62.41*	29.23	*45.97*	7.79	*5.73*	32.19	*48.53*	1.27	29.46
Secondary branch	44	4.45-15.79	*11.89-24.76*	11.44	*18.06*	2.86	*2.01*	13.62	*20.53*	1.19	11.66
**Claw II lengths**											
Primary branch	41	13.48-38.85	*34.41-60.83*	28.76	*45.48*	7.50	*4.76*	30.81	*46.45*	1.24	29.27
Secondary branch	41	5.02-15.56	*13.42-24.40*	11.71	*18.54*	3.06	*1.95*	13.33	*20.10*	1.22	11.90
br	41	*30.97-46.18*	-	*40.91*	-	*3.45*	-	*43.27*	-	-	-
**Claw III lengths**											
Primary branch	41	13.01-39.37	*33.18-59.21*	28.66	*45.29*	7.77	*5.21*	31.74	*47.85*	1.26	28.93
Secondary branch	41	5.70-16.21	*14.94-24.08*	11.82	*18.70*	2.91	*1.95*	13.74	*20.71*	1.12	11.98
**Claw IV lengths**											
Primary branch	39	17.12-50.53	*40.75-72.87*	37.08	*58.31*	10.90	*7.69*	46.01	*69.37*	1.40	37.52
Secondary branch	39	13.25-21.42	*17.45-28.87*	15.38	*24.29*	4.35	*2.83*	18.56	*27.98*	1.30	15.53
br	39	*35.14-51.60*	-	*41.89*	-	*3.70*	-	*40.34*	-	-	-

Character	N	Range	Average	SD
Bare diameter (μm)	54	100.72-136.94	114.93	8.97
Full diameter (μm)	54	122.50-158.41	137.52	8.24
Process height (μm)	53	7.03-15.23	11.57	1.87
Process base width (μm)	53	10.54-21.00	15.15	2.22
Process base / height ratio (%)	53	90.79-210.21	133.22	23.19
Number of processes on the egg circumference	53	20-37	26.17	3.97

Supplementary Table S3

CO1 sequence

Haplotype 1:

5'-
ATATTGGAACACTATATTTTATTTTCGGCCTATGGGCTGCTACCGTAGGAACTTCAC
TAAGATTTATTATTCGATCTGAATTAAGACAACCTGGCTCTATTTTAATAGACGATCA
AATATATAATGTAATTGTAACTAGACATGCATTTATTATAATTTTCTTTTTCGTAATAC
CTATTCTTATTGGGGGATTTGGAAACTGACTAGTGCCCTTAATAATCGGAGCCCCA
GATATAGCTTTCCCACGAATAAATAATTTAAGATTCTGACTGCTACCCCCCTCATTC
ATACTAATTACCAGAGGTACAATAAGAGAACAAGGAGCTGGAACAGGATGAACAG
TTTACCCGCCTTTATCCCAATACTTCGCCCATAGTGGACCAAGTGTAGACCTAACTA
TTTTCTCCCTACATATTGCAGGAGTATCATCCATTCTGGGTGCAATTAATTTTATCTC
CACTATTATTAATATACGATCACCAGCAATAAGAATAGAAAATATGCCGCTATTCGT
GTGATCTGTACTAATTACAGCAATCTTACTACTACTTGCCCTACCTGTACTAGCTGG
GGGTATTACAATACTACTACTAGATCGTAATTTTAATACTTCATTCTTTGATCCTGCA
GGAGGGGGAGATCCAATTCTATACCAACACTTATTCTGATTTTT-3'

Haplotype 2:

5'-
ATATTGGAACACTATATTTTATTTTCGGCCTATGGGCTGCTACCGTAGGAACTTCAC
TAAGATTTATTATTCGATCTGAATTAAGACAACCTGGCTCTATTTTAATAGACGATCA
AATATATAATGTAATTGTAACTAGACATGCATTTATTATAATTTTCTTTTTCGTAATAC
CTATTCTTATTGGGGGATTTGGAAACTGACTAGTGCCCTTAATAATCGGAGCCCCA
GATATGGCTTTCCCACGAATAAATAATTTAAGATTCTGACTGCTACCCCCCTCATTC
ATACTAATTACCAGAGGTACAATAAGAGAACAAGGAGCTGGAACAGGATGAACAG
TTTACCCGCCTTTATCCCAATACTTCGCCCATAGTGGACCAAGTGTAGACCTAACTA
TTTTCTCCCTACATATTGCAGGAGTATCATCCATTCTGGGTGCAATTAATTTTATCTC
CACTATTATTAATATACGATCACCAGCAATAAGAATAGAAAATATGCCGCTATTCGT
GTGATCTGTACTAATTACAGCAATCTTACTACTACTTGCCCTACCTGTACTAGCTGG
GGGTATTACAATACTACTACTAGATCGTAATTTTAATACTTCATTCTTTGATCCTGCA
GGGGGGGGAGATCCAATTCTATACCAACACTTATTCTGATTTTT-3'

18S sequence

5'-
TTCTAGAGCTAATACGTGCAATCAGCTTGCTCTCTCGGGAGCAAGCGCAGTTATTA
GAATAAAAACCAATCCGGCCCTCGGGTCGGTACAATTGGTGACTCTGAATAACCG
AAGCGGAGCGCATGGTCTCGTACCGGCGCCAGATCTTTCAAGTGTCTGACTTATCA
GCTTGTTGTTAGGTTATGTTCCTAACAAGGCTTCAACGGGTAACGGGGTATCAGGG
TCCGATACCGGAGAGGGAGCCTGAGAAACGGCTACCACATCCAAGGAAGGCAGC

AGGCGCGCAAATTACCCACTCCCGGCACGGGGAGGTAGTGACGAAAAATAACGAT
GCGAGAGCTCTAAGCTCCTCGTAATCGGAATGGGTACACTTTAAATCCTTTAACGA
GGATCTATTGGAGGGCAAGTCTGGTGCCAGCAGCCGCGGTAATTCCAGCTCCAAT
AGCGTATATTAAAGTTGCTGCGGTTAAAAAGCTCGTAGTTGGATCTGGGTAGCTGG
ATGGATGGTGTGCCTTGCGGTATTACTGTCTGTTCGGCACCACAAGCCGGCCATGT
CTTGTATGCCCTTCACTGGGTGTACTTGGCGACCGGAACGTTTACTTTGAAAAAAT
TAGAGTGCTCAAAGCAGGCGTACGGCCTTGCATAATGGTGCATGGAATAATGGAAT
AGGATCTCGGATTCTTTTTTGTTGGTTTTCGGAACTCGAGGTAATGATTAATAGGAA
CAGACGGGGGCATTCGTATTGCGGCGTTAGAGGTGAAATTCTTGGATCGTCGCAA
GACGAACTACTGCGAAAGCATTTGCCAAGAATGTTTTCATTAATCAAGAACGAAA
GTTAGAGGTTCGAAGGCGATCAGATACCGCCCTAGTTCTAACCATAAACGATGCCA
ACCAGCGATCCCGTCGGTGTTTTTATCATGACTCGACGGGCAGCTTCCGGGAAAC
CAAAGTGCTTAGGTTCCGGGGGAAGTATGGTTGCAAAGCTGAAACTTAAAGGAAT
TGACGGAAGGGCACCACCAGGAGTGGAGCCTGCGGCTTAATTTGACTCAACACG
GGAAAACTTACCAGGCCAGGACACTTTAAGGATTGACAGATTGAGAGCTCTTTCT
TGATTAGGTGGGTGGTGGTGCATGGCCGTTCTTAGTTGGTGGAGCGATTTGTCTGG
TTAATTCCGATAACGAACGAGACTCTAGCCTGCTAAATAGCCAACTGATCCGCAGC
GTCAGTTGCTAAAAAAGCTTCTTAGAGGGACAGGCGGCGTTTAGTCGCACGAGAT
TGAGCAATAACAGGTCTGTGATGCCCTTAGATGTCCTGGGCCGCACGCGCGCTAC
ACTGAAGGGACCAGCGTGCTTAACCTCCTTGGCCGGAAGGCCTGGGGAATCCGAT
TAAACCCCTTCGTGATTGGGATTGAGCTTTGTAATTATCGCTCATGAACGAGGAATT
CCCAGTAAGCGCGAGTCATAAGCTCGCGTTGATTACGTCCCTGCCCTTTGTACACA
CCGCCCGTCGCTACTACCGATTGAATGATTTAGTGAGGTCTTCGGACTGGCTGTCG
AGACTGTCGCAAGACGGTTTCGTACGGTTGGGAAGACGACCAAA-3’

28S sequence

5’-
ACACGGACCAAGGAGTTCAACATGCGAGCTAGTTGTTGGGACTTGAAGCCCGCTA
GCAAAGTGAAAGCAAGACATAGTGTAACGCTGTGTATTGGCGTGATCCCGTTACTT
GGTCTGAAAAGTCCGAGCCGGGCGCATCGCCGGCCCGTCATAAGCTCACGTGGCT
GTGGCGGAGCCTGAGCTCGCACGTTGAGACCCGAAAGATGGTGAACTATGCCTGG
GCAGGATGAAGCCAGGGGAAACTCTGGTGGAGGTCCGTAGCGATTCTGACGTGC
AAATCGATCGTCTGACCTGGGTATAGGGGCGAAAGACTAATCGAACCATCTAGTAG
CTGGTTCCCTCCGAAGTTTCCCTCAGGATAGCTGGCACTCGGAGAACGTAGTCTCT
CCCGGTAAAGCGAATGATTAGAGGCCTTGGGGTCGAAACGACCTTAACCTATTCTC
AAACTTTAAATGGGTGAGAAGTCTGGCTTGCTTAAATGCGAAGCTGAAGTCTGGA
CGTTGGATACGAGCGCCTAGTGGGCCACTTTTGGT-3’

character	*Dac. ovimutans*	*Dac. caldarellai*
Buccal tube length	54.24 µm	52.20 µm
Body length	437.58	420.00
Ventral lamina (*pt*)	*56.90*	*43.00*
Stylet support insertion point (*pt*)	*69.32*	*77.82*
Primary branch length of claw II (*pt*)	*48.95*	*34.77*
br of claw II	*42.34*	*51.53*
Primary branch length of claw IV (*pt*)	*65.69*	*44.71*
br of claw IV	*37.19*	*48.97*

Supplementary Table S5

**CHARACTER**	1	2	3	4	5	6	10	11	12	13	14	15	16	17
Diameter of egg without processes		99.6	90.0		124.0	141.1	128.8	125.7		97.7	93.4	132.8	135.1	134.4
Diameter of egg with processes		123.9	109.2		140.4	154.6	140.0	142.9		122.6	109.2	145.9	151.0	145.1
Process height	9.8	10.6	10.4	9.3	6.1	9.0	7.6	7.8	15.6	14.3	10.5	5.6	9.0	6.0
	8.8	11.7	10.7	10.0	7.6	7.8	7.1	8.4	15.7	14.3	10.4	6.0	10.1	5.5
	6.4	12.8	10.6	8.8	7.0	8.7	7.2	8.7	16.1	15.1	9.5	6.2	8.4	5.9
Process base width	10.9	16.4	14.1	13.6	13.6	14.3	13.0	18.6	10.8	12.9	11.6	12.7	15.6	11.4
	12.0	17.9	13.9	13.9	14.9	13.4	12.2	17.9	8.9	12.6	11.3	12.3	14.2	11.4
	8.3	17.4	15.4	13.8	17.1	14.6	11.6	18.2	10.2	11.6	12.7	13.4	14.1	11.6
Process base/height ratio	*111%*	*155%*	*135%*	*146%*	*224%*	*160%*	*171%*	*239%*	*69%*	*90%*	*110%*	*227%*	*174%*	*191%*
	136%	*154%*	*130%*	*139%*	*197%*	*172%*	*172%*	*215%*	*56%*	*88%*	*109%*	*206%*	*141%*	*206%*
	131%	*136%*	*145%*	*156%*	*245%*	*169%*	*160%*	*209%*	*63%*	*77%*	*134%*	*217%*	*168%*	*197%*
Distance between processes				1.0	2.0	2.7	5.2	2.2	1.4			2.4	3.5	2.8
				0.8	2.3	3.0	6.7	3.2	1.5			2.7	4.9	2.4
					1.6	3.9	6.6	4.3	2.7			2.8	3.8	1.9
Number of processes on the egg circumference		21	20			24			20					

DNA fragment	Primer name	Primer direction	Primer sequence (5'-3')	Source
18S rRNA	18S-Tard84F	Forward	CCGCGAATGGCTCATTAAATCAG	Giribet *et al*. 1996
	18S-9S	Reverse	GATCCTTCCGCAGGTTCACCTAC	
28S rRNA	28S-1274F	Forward	GACCCGTCTTGAAACACGGA	Markmann 2000
	28S-706R	Reverse	CGCCAGTTCTGCTTACC	
COI	LCO1490	Forward	GGTCAACAAATCATAAAGATATTG	Folmer *et al*. 1994
	HCO2198	Reverse	TAAACTTCAGGGTCACCAAAAAATCA	

Step	18S rRNA			28S rRNA			COI		
	Temperature [°C]	Time [min:sec]	Cycles	Temperature [°C]	Time [min:sec]	Cycles	Temperature °C	Time [min:sec]	Cycles
Initial denaturation	95	5:00	1	95	5:00	1	94	05:00	1
Denatureation	-	-	-	-	-	-	94	01:00	5
Annealing	-	-	-	-	-	-	45	01:30	5
Elongation	-	-	-	-	-	-	72	01:30	5
Denaturation	94	0:30	30	94	0:30	30	94	01:00	35
Annealing	53	0:30	30	42	0:30	30	50	01:30	35
Elongation	72	0:30	30	72	0:30	30	72	01:00	35
Final elongation	72	10:00	1	72	10:00	1	72	05:00	1
Source	This study			This study			Michalczyk *et al*. 2012		

Supplementary references:

1. Giribet, G., Carranza, S., Baguña, J., Riutort, M. & Ribera, C. First molecular evidence for the existence of a Tardigrada + Arthropoda clade. *Mol. Biol. Evol.* **13,** 76-84 (1996).

2. Markmann, M. Entwicklung und Anwendung einer 28S rDNA-Sequenzdatenbank zur Aufschlüsselung der Artenvielfalt limnischer Meiobenthosfauna im Hinblick auf den Einsatz moderner Chiptechnologie. *Shaker Verlag GmbH* (2000).

3. Folmer, O., Black, M., Hoeh, W., Lutz, R. & Vrijenhoek, R. DNA primers for amplification of mitochondrial cytochrome c oxidase subunit I from diverse metazoan invertebrates. *Mol. Mar. Biol. Biotechnol*. **3,** 294-299 (1994).

4. Michalczyk, Ł., Wełnicz, W., Frohme M. & Kaczmarek, Ł. Redescription of three *Milnesium* Doyere, 1840 taxa (Tardigrada: Eutardigrada: Milnesiidae), including the nominal species for the genus. *Zootaxa* **3154,** 1-20 (2012).
